# Shaken, not stirred: blue whales show no acoustic response to earthquake events

**DOI:** 10.1098/rsos.220242

**Published:** 2022-07-13

**Authors:** Dawn R. Barlow, Mateo Estrada Jorge, Holger Klinck, Leigh G. Torres

**Affiliations:** ^1^ Geospatial Ecology of Marine Megafauna Lab, Marine Mammal Institute, and Department of Fisheries, Wildlife, and Conservation Sciences, Oregon State University, Newport, Oregon, USA; ^2^ Department of Computer Science and Department of Physics, Oregon State University, Corvallis, Oregon, USA; ^3^ K. Lisa Yang Center for Conservation Bioacoustics, Cornell University, Ithaca, New York, USA; ^4^ Marine Mammal Institute, Department of Fisheries, Wildlife, and Conservation Sciences, Oregon State University, Newport, Oregon, USA

**Keywords:** acoustics, blue whale, disturbance, earthquake, marine mammals, New Zealand

## Abstract

Quantifying how animals respond to disturbance events bears relevance for understanding consequences to population health. We investigate whether blue whales respond acoustically to naturally occurring episodic noise by examining calling before and after earthquakes (27 040 calls, 32 earthquakes; 27 January–29 June 2016). Two vocalization types were evaluated: New Zealand blue whale song and downswept vocalizations ('D calls'). Blue whales did not alter the number of D calls, D call received level or song intensity following earthquakes (paired *t*-tests, *p* > 0.7 for all). Linear models accounting for earthquake strength and proximity revealed significant relationships between change in calling activity surrounding earthquakes and prior calling activity (D calls: *R*^2^ = 0.277, *p* < 0.0001; song: *R*^2^ = 0.080, *p* = 0.028); however, these same relationships were true for ‘null’ periods without earthquakes (D calls: *R*^2^ = 0.262, *p* < 0.0001; song: *R*^2^ = 0.149, *p* = 0.0002), indicating that the pattern is driven by blue whale calling context regardless of earthquake presence. Our findings that blue whales do not respond to episodic natural noise provide context for interpreting documented acoustic responses to anthropogenic noise sources, including shipping traffic and petroleum development, indicating that they potentially evolved tolerance for natural noise sources but not novel noise from anthropogenic origins.

## 1. Introduction

Understanding the response of wild animal populations to reoccurring acute disturbance has ramifications for assessing impacts to population health and survival [1]. Examining animal response to natural episodic disturbance events can therefore provide valuable context for interpreting potential impacts of anthropogenic disturbance events. Across a broad range of taxa, animals demonstrate varied responses to noise [2]. For example, some may respond by altering how they communicate under noisy conditions [3,4], habituate to disruptive sounds [5] or sustain elevated stress levels over time in the presence of chronic noise [6]. Sound is a critically important sensory modality for marine mammals, and acoustic monitoring is an effective approach to study their occurrence and behaviour [7]. Blue whales (*Balaenoptera musculus*) are globally distributed, vocally active marine mammals that overlap in time, space and acoustic frequency with human activity in coastal regions worldwide [8–13]. Importantly, blue whales display behavioural and acoustic responses to noise generated by anthropogenic activities including mid-frequency active sonar used in military exercises [14–16], seismic airguns used for oil and gas exploration [17], underwater explosions and shipping [16]. Documented responses of blue whales to acoustic disturbance include changes in behaviour state such as halting feeding or increasing swim speeds [14], increased calling activity [17], changes in call loudness [16] and cessation of calling [16]. These behavioural changes may bear physiological consequences that have subsequent impacts on population health [18].

**Figure 1.**
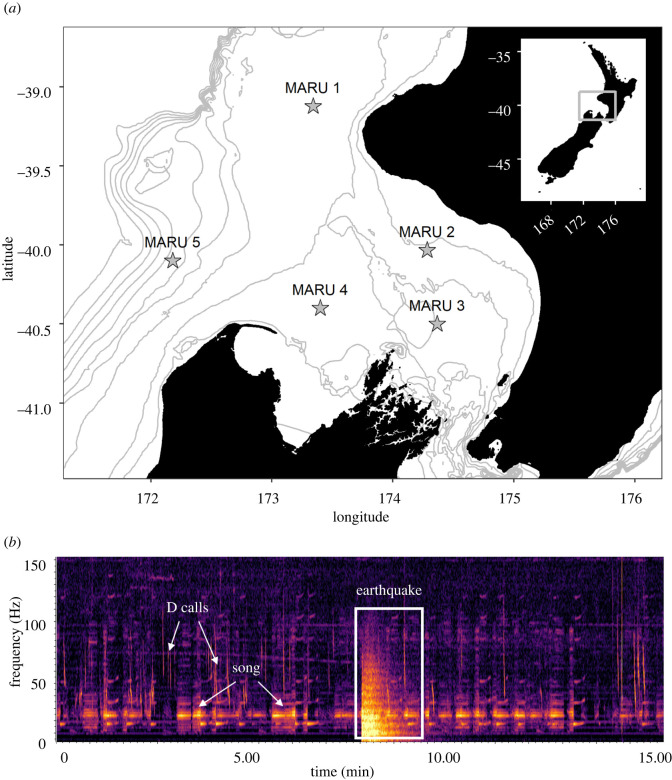
(*a*) Map of hydrophone recording locations, denoted by stars. Location of the study area is indicated by the inset map. Grey lines show bathymetry contours at 50 m increments, from 0 to 500 m; (*b*) Spectrogram of 15 min on 27 April 2016 at MARU4 showing an earthquake, with blue whale song and D calls before and after. Spectrogram visualized with 2048-point fast Fourier transform, Hann window, 90% overlap.

Aotearoa New Zealand and the surrounding waters lie at the intersection of the Australian and Pacific tectonic plates, creating a seismically active region with frequent earthquakes [19,20] that can be detected in the ocean acoustically [21]. The South Taranaki Bight (STB) region, which lies between the North and South Islands, supports an important foraging ground for a unique population of blue whales [22–24] (figure 1). Blue whales are present in the STB year-round, and rely on the region for multiple life-history processes including feeding, nursing and potentially breeding [23]. Given the overlap between blue whale habitat and industrial activities in the STB, including petroleum exploration and vessel traffic [13,22], potential impacts of acoustic disturbance are of increasing conservation concern. Investigating the acoustic response of blue whales to naturally occurring episodic noise from earthquakes can therefore inform and contextualize potential impacts of anthropogenic noise, both in the STB and globally.

**Figure 2.**
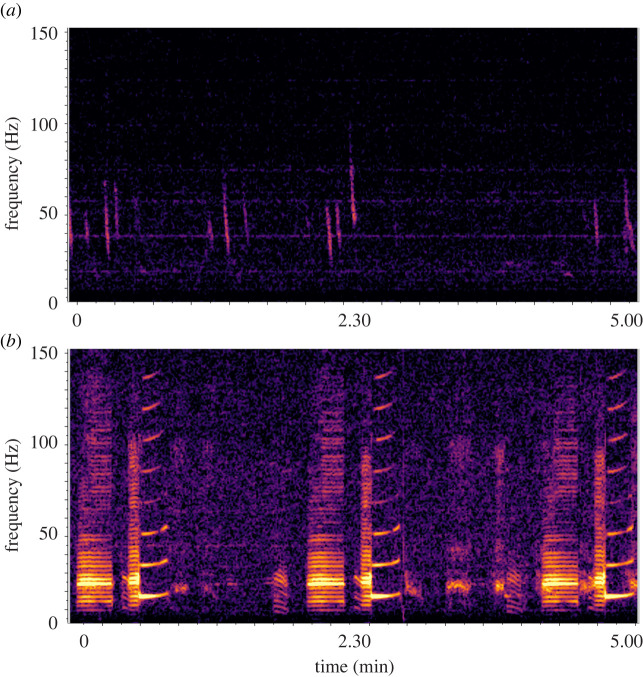
Example spectrograms of the two blue whale call types examined: D calls (*a*) and the New Zealand song (*b*). Spectrograms are configured with a 3072-point fast Fourier transform, Hann window, 50% overlap.

Blue whales produce two main vocalization types: song is produced by males and probably serves a reproductive function, whereas downswept vocalizations known as D calls are social calls produced by both sexes and are associated with foraging behaviour [25,26] (figure 2). Both are frequently detected through passive acoustic monitoring in the STB [23,27]. The frequency band of baleen whale vocalizations including blue whales overlaps with the acoustic signal of earthquakes (figure 1), indicating that whales are capable of detecting and potentially responding to these events [21,28], which may elicit a behavioural or physiological stress response. Considering the frequent occurrence of both blue whale vocalizations [23,27,29] and earthquakes [20,21], the STB region is particularly well suited to examine the potential response of baleen whales to natural acoustic disturbance events. The aim of this study is to describe blue whale calling behaviour before and after earthquakes recorded in the STB, providing a preliminary investigation into call properties surrounding natural disturbance and context for interpreting impacts of anthropogenic noise sources on blue whale acoustic behaviour.

## 2. Methods

### 2.1. Acoustic data collection

Acoustic recordings were collected in the STB as part of a broader effort to understand spatio-temporal occurrence patterns of blue whale vocalizations in the region [23] (figure 1). Five marine autonomous recording units (MARUs) [30] recorded between 27 January and 29 June 2016, each with a flat frequency response (±2.0 dB) in the 15–585 Hz band, and recorded continuously at a 2 kHz sampling rate with a high-pass filter at 10 Hz and low-pass filter at 800 Hz. The recording coverage represents a period with high occurrence of acoustically active blue whales [23].

### 2.2. Earthquakes

Earthquakes were identified from the GeoNet database (https://www.GeoNet.org.nz/), which collates data on known earthquakes identified from a network of seismic sensors around Aotearoa New Zealand. Earthquakes were identified for magnitudes greater than 3.0 that occurred within the study area (between 38–43° S and 172–176° E) during the study period. The magnitude, depth, origin time and epicentre location were extracted for each earthquake. Earthquakes were subset to include only instances where one earthquake was identified in an 8 h period to isolate singular events rather than earthquake swarms or drums. Finally, a subset of 32 individual earthquakes was selected using a stratified-random approach that ensured at least one earthquake was selected for each week to achieve temporal coverage over the recording period.

Earthquakes identified in the GeoNet database that met our criteria were identified in the acoustic recordings based on their timestamp using the acoustic analysis program Raven Pro 1.6 [31]. Raven Pro was used to measure the time the earthquake signal was received and the relative received level (RL) at the hydrophones (measured in dBFS), though we acknowledge that RL at the hydrophones does not necessarily equal RL at the vocalizing whales we detect. Distance to the earthquake epicentre was calculated for each earthquake at all hydrophones where the earthquake signal was detected using the ‘geosphere’ package in R [32].

### 2.3. Blue whale calling

Blue whale calling was examined before and after each earthquake. The analysis was conducted at four temporal scales: 4, 3, 2 and 1 h before and after earthquakes. Therefore, blue whale calls were extracted from recordings at the 4 h scale and subset to shorter temporal windows.

Blue whale D calls (downswept vocalizations approximately 100–20 Hz; figure 2) were identified using an automated detector algorithm optimized for the study system [27]. This spectrogram template correlation detector [33] was constructed using 13 templates to adequately capture the variability among D calls. Putative call detections were compared against the template with the highest spectrogram correlation score, using a detection threshold of 0.80. Once the detector was run on all recording periods of interest, a subsequent manual processing step took place in Raven Pro (5 min spectrograms in the 0–150 Hz bandwidth, 2048-sample Hann window, 50% overlap) to remove false detections and select any calls missed by the detector, thereby ensuring all calls were captured for analysis. The number of D calls was tabulated before and after each earthquake in each temporal window. D call relative RL was computed using the ‘Energy’ measurement in Raven Pro, and mean RL value was calculated before and after each earthquake. Relative RL in dBFS was used as we are interested in the change in calling rather than the absolute RL of calls (and we assume the impact of whale movement on RL within each earthquake analysis period is minimal).

The New Zealand blue whale song (three pulsed calls followed by a tonal call [34], in the 17–24 Hz band [35]; figure 2) occurs often in the region [23], making isolation of individual song calls challenging due to a chorusing effect from overlapping calls. Therefore, a song intensity index was calculated as the ratio of the energy in the predominant song bandwidth (23–24 Hz) relative to selected background frequencies (11, 39 Hz), following a similar approach to [36]. Song intensity was computed at a 1 min resolution, and mean song intensity was calculated before and after each earthquake, within each temporal window.

To examine finer temporal-scale response to earthquakes, number of D calls, D call relative RL and song intensity index were also tabulated at 15 min intervals surrounding each earthquake. This added analysis scale was included to assess whether blue whale calling increased or decreased immediately following the event and subsequently settled out quickly. Mean and standard error for each time interval were calculated across all earthquakes and for all hydrophones and visualized as a time-series for each metric.

### 2.4. Statistical analysis

Paired *t*-tests were used to compare the mean calling activity before and after earthquakes for all call metrics (number of D calls, D call RL and song intensity), and comparisons were visualized using violin plots. Linear models were used to examine whether change in calling (difference between each call metric before and after each earthquake) was related to any earthquake metrics (magnitude, depth, distance to epicentre and relative RL at the hydrophone). Linear models also included day of year to account for seasonal patterns in blue whale calling, number of calls prior to the event to account for ecological context in blue whale calls and hydrophone unit as a factor (table 1). All linear models were run using the ‘stats’ package in R [37].

**Table 1.** Results of linear models examining relationships between earthquake-related metrics and change in blue whale calling. Asterisk indicates statistically significant relationships (*p* < 0.05). Models in rows 1–3 contain earthquake-related predictor variables, whereas models in rows 4–7 only include calling context, day of year and hydrophone unit.

response variable	predictor variables	*F*-statistic	*R*-squared	*p*-value
Δ number of D calls (after – before earthquake)	depth + magnitude + distance to earthquake origin + received energy at hydrophone + number of D calls before* + day of year + factor(hydrophone unit)	5.108	0.277	5.377 × 10^−6^*
Δ D call relative RL (after – before earthquake)	depth + magnitude + distance to earthquake origin + received energy at hydrophone + number of D calls before + day of year + factor(hydrophone unit)	1.673	0.065	0.099
Δ song intensity (after – before earthquake)	depth + magnitude + distance to earthquake origin + received energy at hydrophone + song intensity before* + day of year + factor(hydrophone unit)	2.116	0.080	0.028*
Δ number of D calls (after – before earthquake)	number of D calls before* + day of year + factor(hydrophone unit)	7.032	0.252	2.847 × 10^−6^*
Δ number of D calls (after – before null period)	number of D calls before* + day of year* + factor(hydrophone unit)	7.627	0.262	8.069 × 10^−7^*
Δ song intensity (after – before earthquake)	song intensity before* + day of year + factor(hydrophone unit)	3.540	0.106	0.002*
Δ song intensity (after – before null period)	song intensity before* + day of year* + factor(hydrophone unit)	4.753	0.149	0.0002*

Finally, as a control, an equivalent number of 32 ‘null’ periods were examined during which no earthquakes occurred. Null periods were selected from the time prior to each of the analysed earthquake events, so that they were comparable in terms of the time of year and blue whale presence and behaviour. During null periods, blue whale call metrics were examined in an identical manner as when earthquakes were detected, except before and after periods spanned either side of an arbitrary timestamp rather than an actual earthquake event. Linear models were repeated for these null periods for comparison with the results from the earthquake events (table 1).

## 3. Results

Of the 32 earthquakes examined, the magnitude ranged from 3 to 4.5, distance ranged from 8.02 to 342.2 km from hydrophones, relative RL at the hydrophones ranged between −42.5 and 0 dBFS (electronic supplementary material, figure S1), and duration of acoustic earthquake events was typically 1 min or less. D calls were detected in all temporal windows surrounding earthquakes, yielding 27 040 calls included in the analysis at the 4 h window. Statistical results and observed patterns were consistent across the four temporal windows (electronic supplementary material, figure S2 and table S1); therefore, results from the 2 h window before and after each earthquake are presented here.

**Figure 3.**
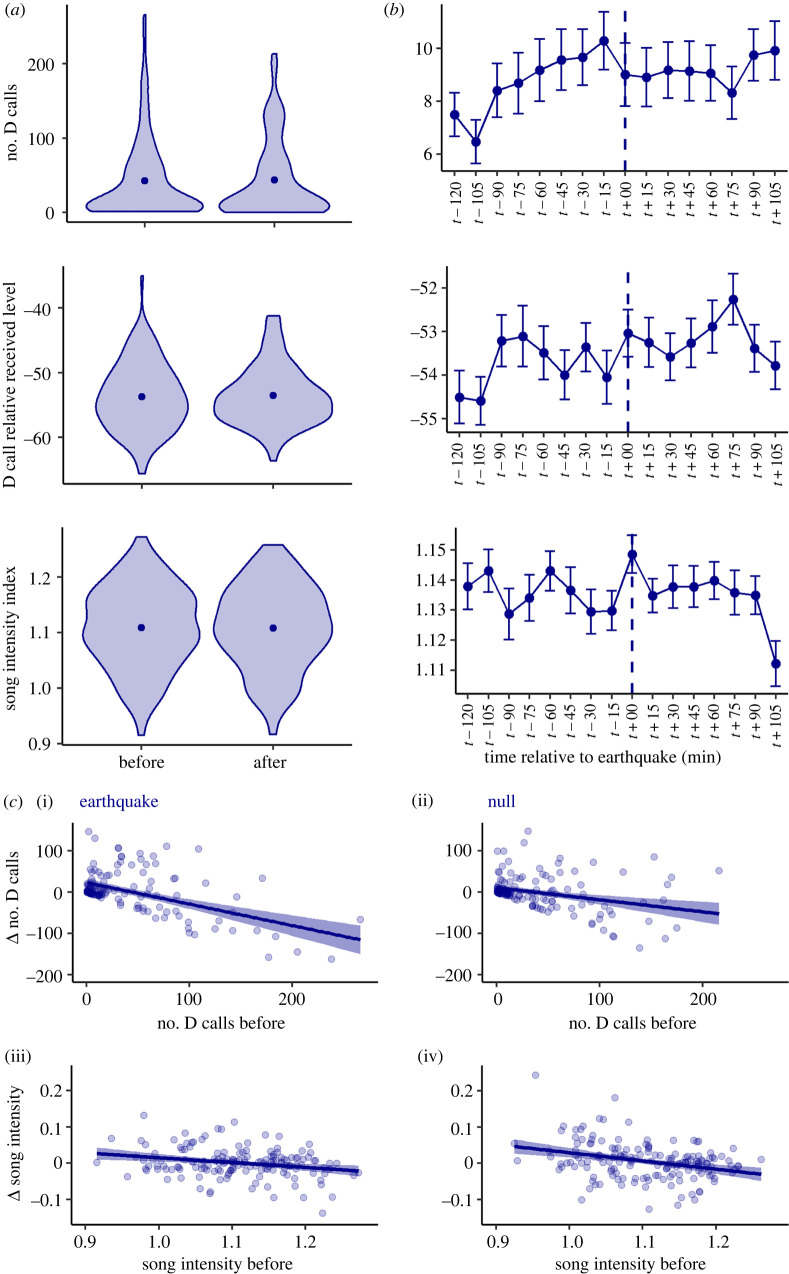
(*a*) Violin plots comparing calling before and after earthquakes. Points indicate mean values. (*b*) Time-series representing each call metric tabulated at a 15 min resolution. Points represent mean values across all earthquake events, vertical lines show standard error and dashed line indicates time of the earthquake. (*c*) Relationships between change in number of D calls and number of D calls before, for earthquake and null events (i, ii), and relationships between change in song intensity and song intensity before, for earthquake and null events (iii, iv). All comparisons shown are for the 2 h temporal window.

There was no visually apparent difference in blue whale calling before and after earthquakes (figure 3*a*). Furthermore, paired *t*-tests revealed no significant difference in mean calling before versus after earthquakes for any call metric (range: *t*-statistic = −0.32–0.05, *p* = 0.74–0.95). While the time-series visualization of the calling metrics at the 15 min resolution revealed fluctuations around earthquake events, the standard error ranges overlapped. Furthermore, fluctuations immediately surrounding earthquakes are not notably different from general fluctuations between 15 min intervals regardless of earthquake presence (figure 3*b*).

Linear models revealed no significant relationships between change in calling and any of the predictor variables for D call RL (table 1). However, models for change in number of D calls and change in song intensity were significant, with number of D calls in the period prior and song intensity in the period prior as significant predictors, respectively. None of the earthquake metrics (magnitude, depth, distance to epicentre and relative RL) were significant predictors of change in blue whale calling in any of the linear models (table 1). The linear model performance for blue whale D call and song intensity metrics with statistical significance did not change notably when the models were computed without earthquake predictor metrics (table 1). Furthermore, the linear models computed using call data from the null periods showed similar model performance and identical patterns as the linear models for earthquake events, indicating these D call and song patterns are unrelated to earthquakes.

Further examination revealed that call metrics yielding significant linear model results (change in number of D calls and change in song intensity) have a negative relationship with the amount of calling in the period prior to an earthquake or null event. Namely, if there was less calling in the period prior, an increase in calling was likely, whereas if there was more calling in the period prior, a decrease in calling was likely. Importantly, this pattern was true for both the earthquake and null periods (figure 3*c*).

## 4. Discussion

We demonstrate that blue whales show no acoustic response to earthquakes, a naturally reoccurring acute noise source, and this finding provides valuable context for how baleen whales respond to anthropogenic noise. The absence of any change in calling activity surrounding earthquakes could indicate acclimation, as blue whales in New Zealand may be accustomed to noise from earthquakes due to their frequent occurrence in the region and over evolutionary time [20,21]. Although we were unable to calculate the absolute RL of earthquake noise at the location of the whales, [21] reported that noise levels associated with a magnitude 7.8 earthquake in Kaikoura, New Zealand, measured 124 dB re 1 µPa in the 12.5 Hz band at a nearby hydrophone. For comparison, the established noise thresholds that constitute harassment of marine mammals are 160 dB for impulsive sources and 120 dB for continuous sources [38]. The earthquakes contained in our dataset are all less than magnitude 4.5 and therefore are probably not as loud as the measured Kaikoura earthquake, thus generally falling below these established thresholds for behavioural response to noise. All detectable sources of noise that overlap in time and frequency with calls can potentially be disruptive to some degree, but given the natural origin and short, episodic duration of earthquake noise, blue whales may have evolved to tolerate this level of occasional disturbance or call masking, at least enough to not elicit an acoustic response. However, cumulative noise from additional sources that are of novel origin and longer duration, e.g. vessel traffic and petroleum exploration, may exceed the threshold beyond which disruption yields more extreme responses [14–17] and potentially bears short- and long-term population-level consequences [18,39].

While we found that earthquakes had no impact on blue whale vocalizations, prior calling context did influence subsequent calling activity. Periods with elevated calling activity (greater number of D calls and higher song intensity index) were significantly related to decreased calling in the subsequent period. This finding may be an artefact of the constraint that calling cannot decrease if there was little calling to begin with. Additionally, in periods with high calling activity, a further increase in calling is unlikely as there may be some degree of call ‘saturation’ already. In other words, in periods with high calling activity, it is more likely for the subsequent period to have less calls irrespective of whether an earthquake occurred. Examination of other temporal properties of the calls (e.g. call unit duration, inter-call interval, repetition rate, etc.) may yield additional insight into these findings on the importance of calling context.

We did not have the ability to localize individual calling whales due to the configuration of the hydrophones in our study, which presents an interesting area for future research into questions we were unable to answer with our data. One way that baleen whales can respond to increasing noise levels is to increase the loudness of their calls, known as the Lombard effect. While this pattern has been described for humpback [40,41], minke [42] and bowhead [43] whales in relation to both natural and anthropogenic noise sources, and for blue whales calling in the presence of ship noise off the coast of California, USA [16], we do not observe this effect in our investigation of blue whales and earthquakes in the STB region. If blue whales in our study did respond to earthquake noise by moving away from the source, this could have been accompanied by an increase in call source level, thereby still yielding no detectable difference in calling. Additionally, the distance to the source of disturbance is probably an important factor in whether blue whales respond, as is the case for humpback whales [44]. The earthquakes included in our study, while all within the STB region, could be beyond the distance threshold at which whales respond. Similarly, the magnitude of the earthquakes in our study period may have been below a threshold strength that would elicit a change in blue whale calling. Furthermore, blue whales show individual-level responses to anthropogenic noise [15]. Therefore, future research into a possible Lombard effect of blue whales in response to noise of both natural and anthropogenic origins, the importance of the distance between a whale and the source of disturbance, and individual-level behavioural responses could provide important insight on the nuanced impacts of noise on blue whale population health.

Our finding that blue whales do not acoustically respond to natural episodic noise is particularly important when considering that they do respond to a range of anthropogenic noise sources [14–17]. The apparent tolerance of blue whales to natural noise contrasts their documented acoustic response to anthropogenic noise, emphasizing the potential negative impacts of these novel sources on the short-term (i.e. communication, mating opportunities) and long-term (i.e. energetic gain, reproductive success) life histories of baleen whales. Hence, our results accentuate the need for careful consideration of cumulative and population-level consequences of anthropogenic disturbance to baleen whales.

## Data Availability

All processed data included in this manuscript and data analysis code are available via the following Figshare digital repository: https://doi.org/10.6084/m9.figshare.20001752. Electronic supplementary material is available online [45].
